# Characterization of the inflammatory cell infiltrate and expression of costimulatory molecules in chronic echinococcus granulosus infection of the human liver

**DOI:** 10.1186/s12879-015-1252-x

**Published:** 2015-11-17

**Authors:** A. Vatankhah, J. Halász, V. Piurkó, T. Barbai, E. Rásó, J. Tímár

**Affiliations:** 2nd Department of Pathology, Semmelweis University, Üllői u. 93, 1091 Budapest, Hungary; Molecular Oncology Research Group, MTA-SE, Budapest, Hungary

**Keywords:** Echinococcosis, Human liver, Immunohistochemistry, Lymphocytes, Cell infiltrate, Macrophage, Dendritic cells, Costimulatory molecules, RT-PCR

## Abstract

**Background:**

The local immune responses to chronic echinococcal infections in various organs are largely unknown. Since the liver is the most frequently involved organ in such infections in human we aimed to characterize the inflammatory as well as immune cell infiltrate around hydatid cysts in the liver and compared to common inflammatory processes of the liver.

**Method:**

Surgical samples from the liver of 21 cystic echinococcosis (CE) patients were studied and the distribution of different types of inflammatory and immune cells were determined by immunohistochemistry. Furthermore, expression levels of costimulatory CTLA4, CD28, CD80 and CD86 molecules were measured at RNA level by PCR. Liver biopsy samples from patients with steatohepatitis (SH, *n* = 11) and chronic hepatitis (CH, *n* = 11) were used as non-inflammatory and chronic inflammatory controls, respectively. The composition and density of the inflammatory and immune cell infiltrates have been compared by using morphometry.

**Results:**

CD3+ T cells predominated the inflammatory infiltrate in all pathological processes, while in CE samples CD20+ B cells, in CH samples CD68+ macrophages were also frequent. Both myeloperoxidase (MPO) + leukocytes and CD68+ macrophages were found to be significantly decreased in CE as compared to either SH or CH samples. Concerning T cell subtypes, only CD8+ T cells were found to be significantly decreased in SH samples. CD1a + dendritic cells were almost completely missing from CE biopsies unlike in any other sample types. There were no differences detected in the mRNA expression of costimulatory molecules except decreased expression of CD28 in CE samples.

**Conclusion:**

In the hydatid lesions of the liver of chronic echinococcal infections T cell-mediated immunity seems to be impaired as compared to other types of chronic inflammatory processes, suggesting an immunosuppressive role for Echinococcus granulosus, which deserve further attentions.

## Background

Cystic echinococcosis (CE) is a globally distributed parasitic disease caused by the larval stage of *Echinococcus granulosus* (Cestoda) [1]. In an intermediate host, the larva (metacestode) can invade various organs where it evolves into a unilocular, fluid-filled (hydatid) cyst [1, 2]. In humans and many other mammalian species, the liver is the major site of CE involvement [2]. The infection induces an immune imbalance on the hepatic tissue, leading to severe destruction of the architecture due to intensive inflammatory infiltrates and formation of fibrosis [2]. It is mostly due to the persistent activation of immune system which imposes unfavorable changes on normal homeostasis of the organ, however the parasite can usually evade the host defense and the infection becomes chronic [2].

Normal liver immune homeostasis is sustained by different cells which reside the organ [3]. Inflammatory responses to an injury are characterized by dynamic changes in cellular architecture of the liver [3]. A widely accepted scenario defines the role of T helper (Th) 1 cytokines which induce classical activation of macrophages (MΦs) and infiltration of non-resident polymorphonuclear (PMN) cells in acute responses [4]. Chronic inflammation is associated with alternatively activated MΦs by Th2 cytokines which result in transformation of hepatic stellate cells (HSCs) and fibrosis [4, 5]. Regulatory T cells (T_reg_), as resident cells with immunosuppressive traits, may have role in maintaining the physiological stability of the organ [6]. Signaling pathway through CD80 (B7-1)/CD86 (B7-2)-CD28/CTLA4 costimulatory molecules has been proposed to have a crucial role in regulation of the T cell-mediated immunity [7, 8]. The modal shift in cytokine profiles from Th1 to Th2 is known as a hallmark of immune responses to tissue-dwelling helminths [2, 9]. It may facilitate the parasite survival in the host but results in chronic granulomatous reactions and fibrosis [2]. Nevertheless, little is known about the arrangement of resident and non-resident cells in chronic responses to hydatid cyst in the human liver.

Characterization of inflammatory infiltrates in the chronic CE has been assessed in only a few studies using experimentally or naturally infected animals. Results of these studies should be cautiously extended to humans due to the primary impact of host-specificity on the infection pathogenesis [10, 11]. Indeed, the parasite provokes less severe pathological alterations in its normal hosts (mostly herbivores in both domestic and sylvatic life cycles) than in incidental (humans) or aberrant (laboratory animals) hosts [11, 12]. Normal hosts also show different susceptibility to the infection [13]. The existing knowledge of human immune reactions to the chronic CE has been merely derived from peripheral blood mononuclear cells (PBMCs) assays and serological measurements. Cytokine profiling analyses have illustrated a contradictory scheme of the T cell response as concurrent activity of interleukin [4]-2, interferon gamma (IFN-γ), tumor necrosis factor alpha (TNF-α), IL-4, IL-5, IL-6 and IL-12 can be detected in patients [2, 14]. Results of separated studies showed traceable levels of TNF-α, IFN-γ, IL-4, IL-5, IL-6, IL-10, IL-12, IL-13, IL-16, IL-17A and IL-18 in the sera of hydatid patients, although IL-6, IL-17A and IFN-γ had impaired activity in those with relapsed disease [15–17]. Serological methods may only indicate the overall systemic immunity which can be interfered by temporal changes and many factors such as genetic traits, physiological status and immune responsiveness of the individual, as well as the presence of cross-reactive immune components and the localization of the cyst [16, 18–21]. Besides, Results of *in vitro* studies provide indirect evidence of the human immune response to CE, which may differ under experimental conditions. As an instance, exposure of human PBMCs to the parasite specific antigen B and its recombinant subunit prompted skewed Th2 and Th1 cytokines, respectively [22–24]. Whereas, hydatid crude antigen induced expression of IL-4, IL-5, IL-6 and IFN-γ in PBMCs isolated from patients and showed correlation with the infection status [25–27]. Collectively, these findings suggest the concomitant activation of Th1 and Th2 in the chronic CE. The expression of IL-10 may indicate the presence of T_reg_-mediated immunoregulatory mechanisms [28, 29]. Such a presumption shows significant disparities between hydatid-induced immune profiles and the scenario of cellular activities in the liver chronic inflammations, thus may not yet implicate the characteristics of organ-specific cellular responses in chronic CE of the human liver.

It can be hypothesized that the cellular composition of inflammatory infiltrates around the hydatid lesion in the human liver is likely governed by intrinsic mechanisms that are specific to the organ and are directly activated in response to a massive amount of parasite antigens, however it has not been accurately described yet. Accordingly, this study was aimed to characterize the cellular populations, which infiltrates the liver in chronic human CE. An immunohistochemical analysis was herein performed to quantify the inflammatory cell infiltrates in liver biopsies of CE patient by using a panel of mono/polyclonal antibodies against CD1a, CD20, CD68, myeloperoxidase (MPO), smooth muscle actin-α (α-SMA), CD3, CD4, CD8, and FOXP3. To asses T cell mediated functions further at the periphery of hydatid cyst, mRNA expression level of costimulatory molecules (CTLA4, CD28, CD80 and CD86) was quantified by RT-PCR. CE patient cohort was compared to those with steatosis (SH) and chronic inflammation of the liver (CH).

## Methods

### Patients and sample preparation

Formalin-fixed, paraffin-embedded tissue samples of the liver from 21 surgically operated CE patients, 14 females (66.7 %) and 7 males (33.3 %), between 5 to 79 years of age were obtained from the archives of the 2^nd^ Department of Pathology at Semmelweis University, Budapest, Hungary. All samples had been prepared from total or subtotal pericystectomy materials showing cyst lumen, fibrous capsule and liver parenchyma adjacent to the parasitic lesion. Biopsies from 11 patients with CH, 4 females (36.4 %) and 7 males (63.6 %) between 2 months to 63 years of age and 11 patients with SH, 7 males (63.6 %) and 4 females (36.4 %) between 12 to 75 years of age were also included. This study obtained licence from the institutional review board of the Semmelweis University (TUKEB15/2008).

### Immunohistochemistry

Tissue sections of 2.5 μm thickness were prepared using Thermo Scientific Finesse E^+^ microtome (Thermo Scientific, Waltham, MA, USA). Some sections were mounted on microscope slides and were stained by hematoxylin-eosin method. Immunohistochemistry (IHC) was performed by using BenchMark XT automated slide preparation system (Ventana Medical Systems, Tucson, AZ, USA) and assays were run by applying UltraView DAB detection kit (Ventana Medical Systems). The kit was optimized regarding an indirect IHC protocol which was based on heat-induced epitope retrieval (HIER) method, using citrate-EDTA buffer (10 mM citric-acid, 2 mM EDTA, 0.05 % Tween20, pH: 6.2) for unmasking calcium ions-masked tissue and avidin/biotin solution for reducing background or unspecific staining. 2,3-diamino-benzidine, copper sulphate and hematoxylin II were used as chromogen, post-chromogenic enhancement and counterstain agents, respectively. All specimens were examined with a panel of mono/polyclonal antibodies against human cell surface markers corresponding designated cell populations.

### mRNA isolation and quantification

Total RNA was isolated using High-Pure RNA Paraffin kit (Roche Diagnostics, Penzberg, Germany). TURBO DNA-Free kit (Ambion, Austin, TX, USA) was used to remove the contaminating DNA from subsequent isolates. Elimination of DNA was controlled by a conventional PCR optimized for amplification the housekeeping gene β-actin, using 2 μl of each isolate mixed with 12.5 μl dNTPs/Taq polymerase (Fermentas Life Sciences, Pittsburgh, PA, USA), 2.5–2.5 μl of the appropriate primer pair (0.5 μM), 0.5 μl MgCl_2_ and nuclease-free water (nfH_2_O) to the total volume of 23 μl. Total RNA was reverse transcribed to cDNA with oligo-dT and random hexamer primers using TaqMan reverse transcription reagents (Life Technologies, Carlsbad, CA, USA) as per manufacturer’s instructions. In brief, 8 μl of purified total RNA with 1 μl dNTP (10 mM) and 1 μl oligodT and random hexamer were mixed and were incubated for 10 min at 70 °C in a thermal cycler. Tubes were chilled on ice for 2 min and 2 μl reverse transcriptase buffer, 1 μl M-MLV reverse transcriptase and 0.5 μl RNase inhibitor (all provided by M-MLV Reverse Transcriptase Kit, SIGMA) with 6.5 μl nfH_2_O were added to each tube. This procedure was continued by one hour incubation at 37 °C in a thermal cycler and products were tested by β-actin PCR for presence of cDNA. Primer pairs were designed specific for one splicing variant (transcript variant 1 of each molecule mRNA sequence) and oligonucleotides:

- for CTLA4, sense 5′-TGGCTTGCCTTGGATTTCAG-3′ and
  - antisense 5′-GGAATCATCTAGGAAGGTCAACTC-3′,
- for CD28, sense5′-AAGCATTACCAGCCCTATG-3′ and
  - antisense 5′-AGAGCAGTGATATTGAGCAGATG-3′,
- forCD80, sense5′-CCTGGTTGGAAAATGGAGAA-3′and
  - antisense5′-AGGAAAATGCTCTTGCTTGG-3′,
- for CD86, sense 5′-TGTCAGTGCTTGCTAACTTCAG-3′ and
  - antisense 5′-TGGTCATATTGCTCGTAACATCAG-3′,

were synthesized by Integrated DNA Technologies (IDT, Coralville, IA, USA) upon order. The specificity of primers was evaluated by employing a PCR (as above) and amplified products were separated on a 3 % agarose gel. After ethidium bromide staining, reactive bands were visualized by a transluminator (Ultraviolet Light Box, GBox, SYNGENE, Synoptic Group, UK) and then were excised from the gel and cDNA was recovered by using EZ-10 Speed Column DNA kit (Bio Basic, Ontario, Canada). The DNA sequences were determined by BigDye Terminator v1.1 Cycle Sequence kit (Applied Biosystems, Foster City, CA, USA), and were matched to corresponding molecules by a BLAST search.

Real-Time PCR was carried out using iQ SYBR-Green Supermix kit (Bio-Rad). All reactions were in triplicate with cycling parameters as: 95 °C for 3 min, 50 cycles (95 °C for 15 s, 56 °C for 30 s and 72 °C for 40 s), followed by a melting curve rising from 56 to 95 °C and a cooling cycle at 37 °C for 10 s. Starting quantities were defined on the basis of standard fivefold dilution series (1X-625X) were prepared with control cDNA of human colon cancer (cell line A431, Molecular Pathology Lab, 2^nd^ Department of Pathology, Semmelweis University, Budapest, Hungary) and all measurements were normalized with housekeeping β-actin (background gene) starting quantities from the same cDNA samples. Standard error in technical triplicate ranged from 0.9 to 3.0 % (average: 2.2 %) and nfH_2_O was used as the no-template control.

### Data analysis

Results of IHC test were evaluated by applying a semi-quantitative assessment: each slide was observed with a light microscope for three times at x400 magnification and about 10 fields were randomly selected in each round. Thus, the number of immunolabeled cells was counted in 30 fields under a fixed focus for each slide and value of median for total count of each identified cell phenotype was scored and assigned to groups as follows:

Data were expressed as mean ± standard deviation (SD) and Analysis of Variance (ANOVA) test was used for differences between groups. Spearman’s rank correlation coefficient (Rho) was calculated for dependence between quantified mRNA level of costimulatory molecules and corresponding immunostained cell phenotypes in biopsy samples. Relative frequency (RF) of each cell type was calculated as the mean ratio (%) of a cells phenotype (mean) in total number of all counted cells for each sample group. Values *p* < 0.05 were regarded as statistically significant.

## Results

### Histology

Tissue damage and fibrosis were observed in all samples obtained from CE patients. Inflammatory cell infiltrates were detected in all CE samples around the fibrous capsule mainly consisting of fibroblast-like cells along with infiltrating lymphocytes, occasional polymorphonuclear cells and monocytes. Hydatid cysts with partial calcification were observed in 8 % (5 of 21) of patients. Due to the presence of protoscolices or hooklets in histology sections, cysts were identified as fertile in all patients. Ductular proliferation and hydatid cyst rupture into biliary tracts were observed in 4 patients (19 %) (Table 1). Hepatocytes with large lipid droplets in their cytoplasm were detected in all SH samples. Ductular reaction (*n* = 8, 73 %) and slight tissue remodeling and mild accumulation of connective tissue fibers (*n* = 5, 45 %) were also observed in some SH biopsies. Pericellular scar and septal fibrosis along with macrovesicular steatosis and inflammatory nodules were detected in 2 (18 %) SH patients. Iron staining demonstrated the present of hemosiderin in ~30 % of Kupffer cells in these biopsies. Besides, apoptotic hepatocytes were observed among normal cells in the parenchyma. Histopathological examination of CH biopsies showed a range of different alterations in the liver from mild infiltration of inflammatory cells and slight remodeling in the lobular structures of portal areas to septal fibrosis. Portal fibrosis was observed in 64 % (*n* = 7) of these CH biopsies (Table 1).

**Table 1:** Pathology of the liver biopsies of patients with cystic echinococcosis (CE, *n* = 21), steatosis (SH, *n* = 11) and chronic hepatitis (CH, *n* = 11)

Sample Groups	Range of Age (Year)	Sex	Pathology					
		Male (n)	Female (n)	Intrabiliary Rupture (n)	Portal Fibrosis (n)	Periportal Fibrosis (n)	Septal Fibrosis (n)	Ductular Reaction (n)
CE	41 ± 19.31	7	14	4	10	2	1	-
SH	26 ± 22.31	7	4	-	5	-	2	8
CH	26 ± 19.31	7	4	-	7	-	1	7

### Quantitative evaluation of inflammatory cell densities

**Fig. 1:**
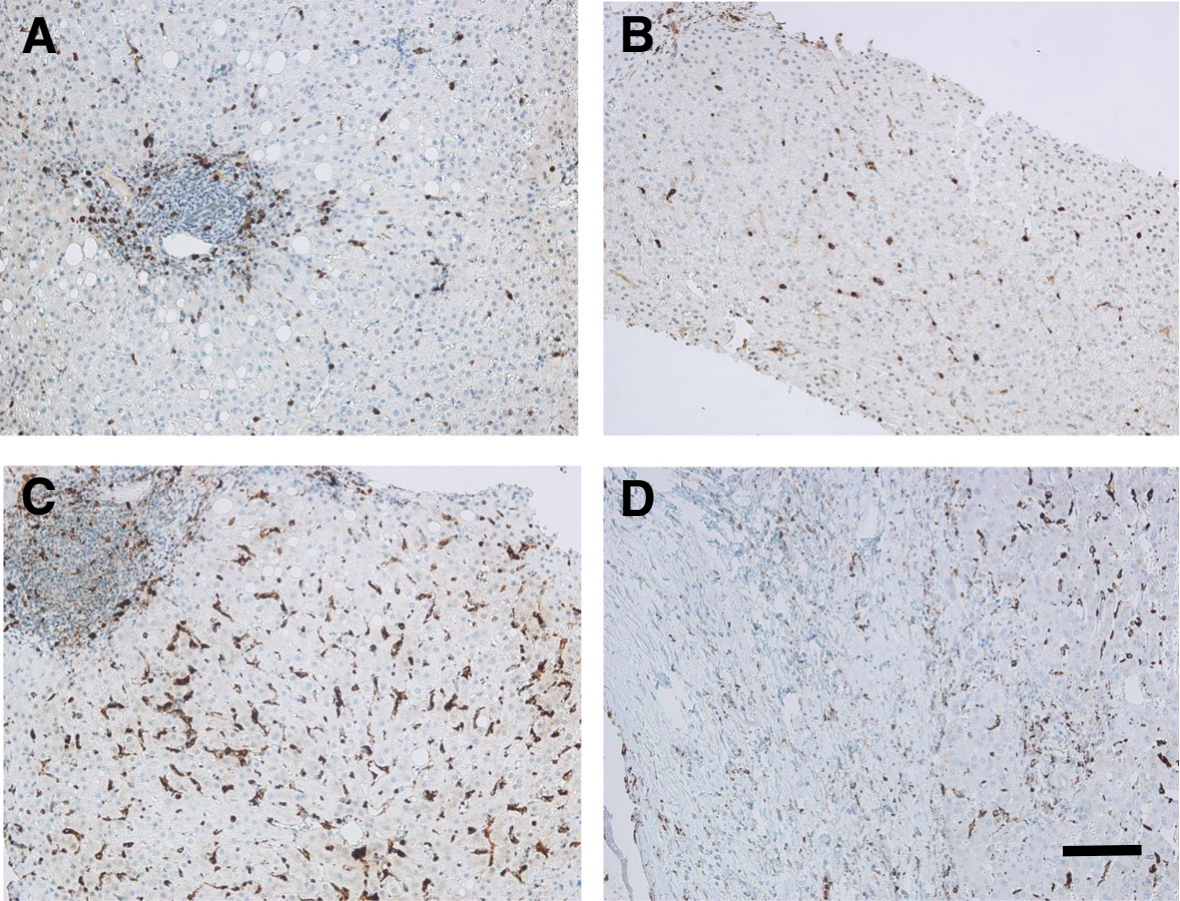
Localization of leukocytes and macrophages in liver biopsies. **a**/**b** Localization of neutrophil leukocytes as detected by MPO immunohistochemistry in CH (**a**) and CE (**b**) samples. Note the decreased density in CE biopsy. **c**/**d** Localization of macrophages by CD68 immunchemistry in CH (**c**) and CE (**d**) biopsies. Note the significant infiltration of macrophages in CH (**c**) and decreased presence in CE sample (**d**). Bar: 100 μm

In all CE biopsies, MPO^+^ cells were absent or were scanty, localized mostly within sinusoidal spaces of the liver. The density of these cells (Fig. 1) scattered within inflammatory areas of the parenchyma in SH and CH was higher than that in CE, although the statistical analysis only confirmed the significant difference between CE and CH in this regard, *p* = 0.01 (Table 2).

**Table 2:** Density of inflammatory cells of the liver in cystic echinococcosis, steatosis and chronic hepatitis biopsy samples

	CE	SH	CH	p Value		
Marker	Cells/field (mean ± SD)	Cells/field (mean ± SD)	Cells/field (mean ± SD)	CE vs. SH	CE vs. CH	SH vs. CH
**CD1a**	**0 ± 1.1**	**13 ± 11.85**	**8 ± 9.63**	**0.00001**	**0.006**	**0.5**
CD3	265 ± 138.66	232 ± 198.11	241 ± 143.89	0.9	0.9	0.9
CD4	29 ± 18.94	24 ± 22.86	57 ± 46.16	0.1	0.1	0.1
**CD8**	**20 ± 50.61**	**17 ± 26.62**	**36 ± 23.27**	**1**	**0.02**	**0.1**
FOXP3	4 ± 4.53	10 ± 8.64	20 ± 29.87	0.1	0.1	0.1
CD20	230 ± 174.86	50 ± 53.78	140 ± 128.50	0.06	0.06	0.06
**CD68**	**101 ± 147.13**	**108 ± 77.47**	**258 ± 107.82**	**1**	**0.0006**	**0.05**
**MPO**	**35 ± 30.89**	**65 ± 28.82**	**133 ± 118.14**	**0.06**	**0.01**	**1**
**α-SMA**	**537 ± 75.63**	**394 ± 104.19**	**364 ± 130.97**	**0.001**	**0.001**	**1**

*Data* are mean number of immunostained cells (M) ± SD in CE (cystic echinococcosis), SH (Steatosis), and CH (chronic hepatitis) biopsies as determined in Materials and Methods section. Value of significance (p) has been exhibited for differences between sample groups in pair

*MPO* Myeloperoxidase (presented in bold)

**Fig. 2:**
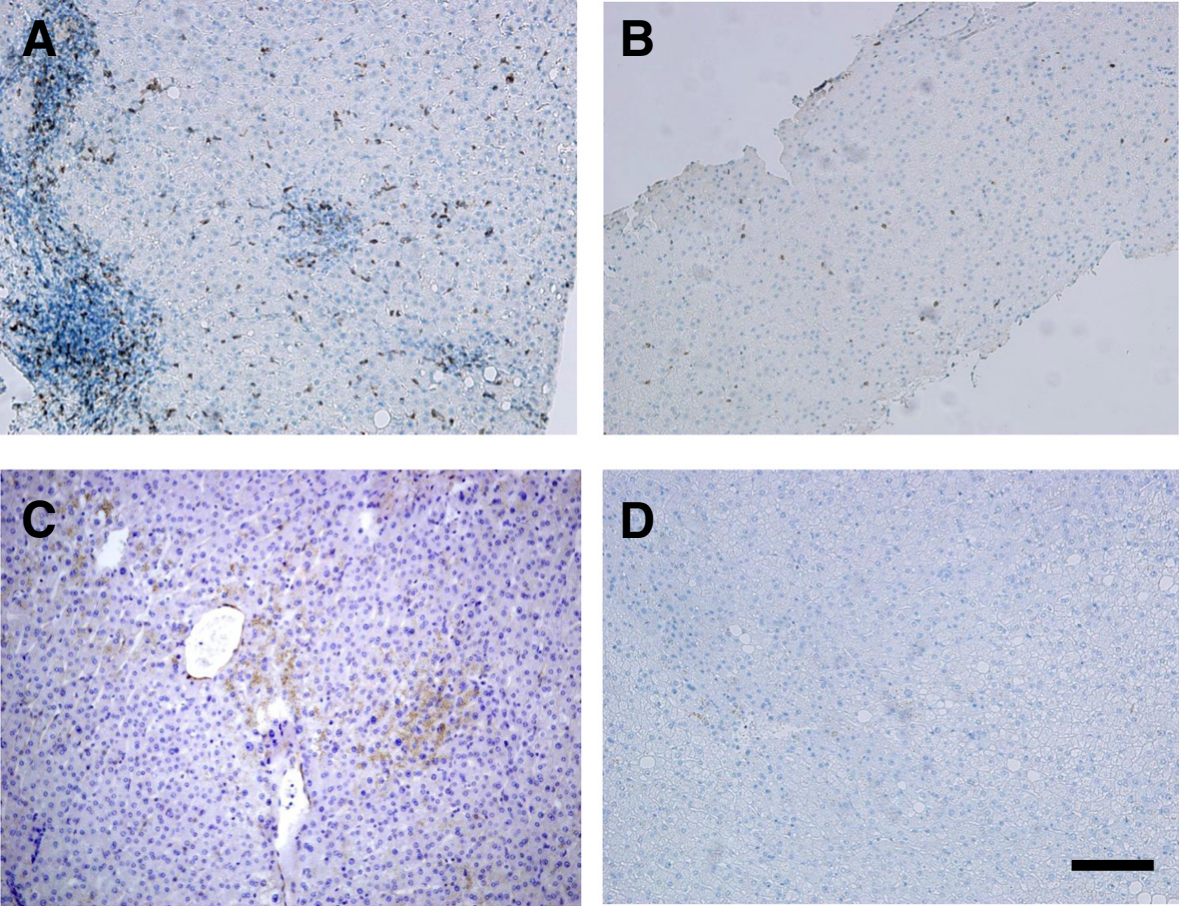
Localization of cytotoxic T cells and dendritic cells in liver biopsies. **a**/**b** Identification of cytotoxic T cells by CD8 immunohistochemistry in CH (**a**) and CE (**b**) samples. Note the significant portal and lobular presence of CD8 + T cells in CH (**c**) and a decreased infiltration in CE sample (**b**). **c**/**d** Identification of dendritic cells in liver biopsies using CD1a immunohistochemistry. Note the complete absence of CD1a + cells in CE (**d**) as compared to CH (**c**) sample. Bar: 100 μm

T cells predominantly consisted of CD3^+^ cells in all liver biopsies. These cells were mostly observed as densely accumulated clusters around the pericyst in CE biopsies. There was no significant difference between the sample groups according to the number of labeled CD3^+^ T lymphocytes. In CE livers, CD4^+^ and CD8^+^ cells were absent or had very low to low counts at the site of injury characterized by small cellular clusters of adhered to the pericystic adventitia as well as scattered cells throughout the parenchyma. CE liver biopsies from 6 patients (29 %) lacked CD8^+^ cells and the amount of these cells was relatively moderate at the periphery of hydatid lesion in two patients (9.5 %). There was no significant difference according to the density CD8^+^ cells between CE and SH, whereas such a value was found significantly higher in CH biopsies, *p* = 0.02 (Fig. 2) (Table 2). Aggregation of CD4^+^ T cells around the pericyst was scored as very low to low (from 0 to max 67 cells/filed, mean: 29 ± 18.94 cells/field) with less disparity regarding the number of infiltrated cells between individual samples and was not significantly different than that in SH and CH biopsies. Cells with FOXP3^+^ phenotypes were absent or had very low score in all sample groups (Table 2).

B lymphocytes comprised the second largest cellular population and were identified by expression of CD20 surrounding the hydatid lesion. The amount of CD20^+^ cells quantified in CE livers was higher than SH and CH, however this difference was not statistically significant. In all samples, these cells formed focally aggregated clusters adjacent to the scar tissue. Number of CD68^+^ cells was found highly variable in CE livers, ranged from 0 (in one sample) to ≥500 (in two samples). The density of CD68^+^ macrophages in the inflammatory infiltrate of CH livers was significantly higher than that in CE and SH samples, *p* < 0.001 (Fig. 1) (Table 2).

Rare scattered CD1a^+^ cells were observed throughout the parenchyma SH (13 ± 11.85 cells/field) and CH (8 ± 9.63 cells/field) livers, whereas they were completely absent in CE biopsies (Fig. 2). No difference was found between SH and CH regarding the number of CD1a^+^ cells in the inflammatory milieu of the liver (Fig. 2) (Table 2).

Among the perihydatid cells α-SMA^+^ myofibroblasts were the most frequent cell type constituted the non-parenchymal cell population in all biopsies. Relative frequency of these cells was higher in CE and SH than that in CH, p ≤ 0.05. There was no significant difference between CE and SH groups according to the relative frequency of CD68^+^ cells in inflammatory infiltrates of the liver, but this value was significantly higher in CH samples, *p* < 0.05. On the contrary, CD20^+^ cells showed the highest frequency in CE compared to SH and CH, *p* < 0.05. Altogether, CD3+ T lymphocytes were the most frequent subtype of T cells and showed similar pattern in all biopsies. Other activation markers of T cells were detected on much smaller proportion of infiltrated cells within inflammatory regions of the liver in all groups (Fig. 2).

### Expression level of costimulatory molecules

**Fig. 3:**
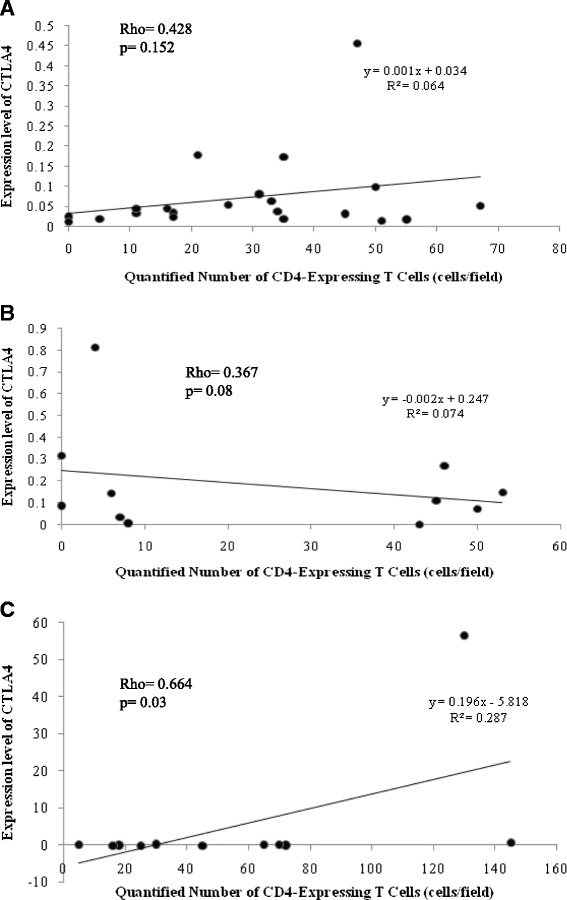
Correlation between quantity of CD4^+^T cells and mRNA expression level of costimulatory CTLA4 molecule in liver samples. **a** = CE, **b** = SH, **c** = CH biopsies. Statistical analysis identified only significant correlation between the two factors in CH samples (**c**)

Antigen presenting cells associated costimulatory molecules (CD80-CD86) had high expression level in all biopsies. The expression of CD80 and CD86 at the mRNA level in CH biopsies was slightly higher than other two groups but differences were not significant (Table 3) (Fig. 3). CTLA4 was highly expressed at the mRNA level in CH livers that showed 435-fold and 73-fold increase compared to CE and SH, respectively. Expression level of CTLA4 in SH was also higher (6-fold) than CE. However the average level of CTLA4 expression was not significantly different between all groups more likely due to the wide range of inter-sample variations. Expression of CD28 had a remarkable decrease in CE livers compared to other sample groups and such a difference was statistically significant (SH/CE = 2171 fold, *p* = 0.0001), (CH/CE = 6191 fold, *p* < 0.00001) (Table 3).

**Table 3:** mRNA expression levels of costimulatory molecules in inflammatory infiltrates of the liver in cystic echinococcosis, steatosis and chronic hepatitis biopsy samples

Costimulatory Molecules	CE	SH	CH		P Value	
	Mean ± SD	Mean ± SD	Mean ± SD	CE vs SH	CE vs CH	SH vs CH
CTLA4	0.073 ± 0.099	0.182 ± 0.233	5.293 ± 16.949	0.1	0.07	0.1
**CD28**	**0.087 ± 0.018**	**24.361 ± 22.860**	**71.128 ± 60.365**	**0.0001**	**<0.00001**	**0.1**
CD80	50.779 ± 67.682	54.731 ± 74.778	67.712 ± 98.575	0.5	0.1	0.5
CD86	61.528 ± 117.973	27.526 ± 33.573	62.451 ± 89.896	0.2	0.5	0.5

Expression of costimulatory molecules was quantified by calculating the ratio of target mRNA level to a background houskeeping β-actin mRNA and was defined as mean ± SD for three sample groups: cystic echinococcosis (CE), steatosis (SH) and chronic hepatitis (CH) (presented in bold)

The ratio of CD28/CTLA4 in CE samples was 0.3 showing a slightly higher prevalence of CTLA4 expression around the hydatid lesion. Such a ratio in CH samples was found to be 4, and was the highest (CD28/CTLA4 = 93) in SH biopsies. On the other hand, the level of CD80 mRNA was higher than CD86 at CD80/CD86 ratios of 1 and 2 in SH and CE, respectively, but this ratio reversed in CH biopsies (CD80/CD86 = 0.7).

Expression of CTLA4 and CD28 had no significant correlation with the density of various phenotype markers (CD3^+^, CD4^+^, CD8^+^, and FOXP3^+^) in CE and SH samples. In CH livers, expression of CTLA4 was positively correlated with the quantity of CD4^+^ cells in each individuals, Rho = 0.664, *p* = 0.03 (Fig. 3).

**Fig. 4:**
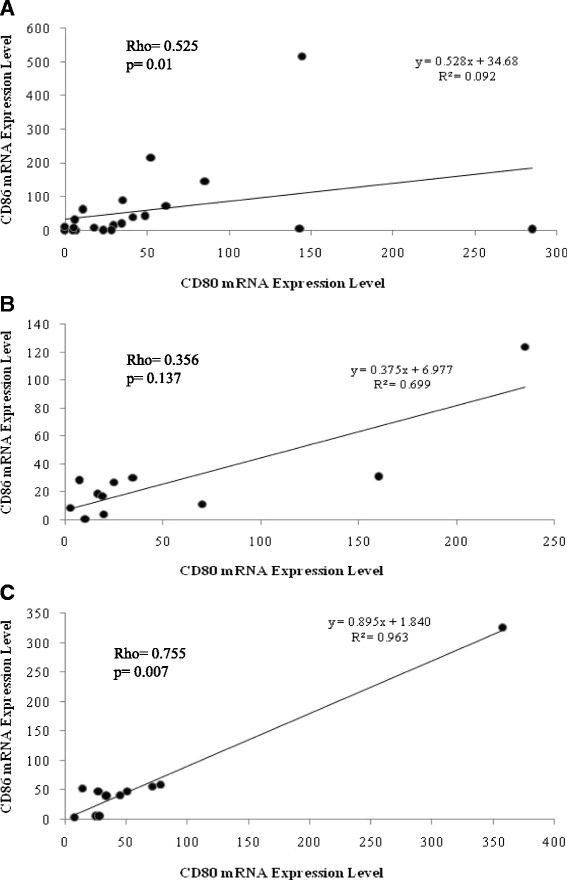
Correlation between CD86 and CD80 mRNA expression levels in liver biopsies. Statistical analysis identified significant correlation in CE (**a**) and CH (**c**) samples where no such correlation was detected in SH materials (**b**)

Expression of CD80 and CD86 measured in CE and CH livers was significantly correlated each other (Rho = 0.525, *p* = 0.01 and Rho = 0.755, *p* = 0.007, respectively), while this correlation was not significant in SH (Fig. 4).

## Discussion

Our data indicated an overall impairment of T cell activities in the liver of patients with chronic CE. As novel data, we have shown the expression pattern of costimulatory molecules in the inflammatory infiltrate surrounding chronic hydatid lesions in human liver. According to our results, the expression of CTLA4 mRNA was very low in all sample groups. Similarly, CD28 mRNA had a significantly lower level in CE than that in SH and CH. Signal transduction through CD28, which is constitutively expressed on both CD4^+^ and CD8^+^ subtypes, is essential for the T cell activation [7]. To reduce the magnitude of immune reactions, some CD4^+^ T cells express CTLA4 which has downregulatory effect on CD28 [30]. The function of CTLA4 is associated with the T_reg_ cells activity and its presence is dependent on expression of FOXP3 [30]. The expression of CTLA4 mRNA was significantly correlated with the presence of CD4^+^ cells only in CH biopsies; however this correlation between CTLA4 and FOXP3 was insignificant in all sample groups. The phenotypic change of T cells to CD28^−^ subtypes has been reported in infections, autoimmune diseases and cancer [31–34]. The role of costimulatory molecules in CE has been barely defined, but hydatid fluid antigen seems to induce diminished expression of CD28 in T cell cultures [35]. Insignificant expression of CD28 and CTLA4 mRNA measured in this study could suggest the poor activity of T cell-dependent mechanisms at the periphery of hydatid cyst in human chronic infection, probably due to the clonal deletion of specifically activated T cells [36].

Immunohistochemistry also revealed that CD3^+^ cells were the most frequent T cells infiltrated the inflammatory milieu of the liver in all sample groups. In contrast, CD4^+^ and CD8^+^ subpopulations were significantly outnumbered at the periphery of hydatid lesions in CE biopsies at a CD4^+^/CD8^+^ ratio of 10.30. This ratio was 0.85 and 1.29 for SH and CH biopsies, respectively. T cells dominated by the CD3^+^ subtype reported in the pericystic adventitia of the hepatic cysts in sheep, while CD8^+^ and CD4^+^ cells were the most frequent populations in cattle with progressive and regressive cysts, respectively [29, 37]. Intriguingly, 40–70 % of cysts in humans and sheep are fertile, while 70–100 % of cysts in cattle have degenerated or undeveloped structures [38–42]. Altogether these findings along with outcomes of the present study suggest the impact of the host susceptibility of the composition of T lymphocytes which are locally activated in response to the liver hydatid infection. Besides, the density of CD8^+^ cells was found to be different in three sample groups. Although such a difference was not significant between CE and SH, it could imply the prognostic value of CD8-dependent T lymphocyte activity in chronic inflammations of the liver with different etiology. Presumably, predominance of CD3^+^ immunostained cells with weak presence of CD4^+^ and CD8^+^ subtypes in CE biopsies also highlighted the role CD3^+^CD56^+^ natural killer T cells in the hydatid-induced chronic inflammation of the liver, which is worth further investigation [43].

Abundant number of B cells expressing CD20 observed at the periphery of hydatid lesions, demonstrated significant activity of adaptive immune system. In normal conditions, B cells comprise a very low proportion of the liver resident cells, but they infiltrate the parenchyma during the inflammatory response [44]. The humoral immune response to hydatid cyst in human has been studied by applying indirect measurements [2]. Therefore, our data could denote the importance of B cells in immune functions which are locally activated against the parasite in the liver.

Furthermore, results of this study demonstrated the inadequate involvement of PMN cells and MΦs in chronic responses to CE in the human liver. Evidently, the antibody-dependent cell mediated cytotoxicity (ADCC) is an important defense mechanism primarily activated against the chronic phase of helminth infections [2]. During the early phase of infection, type 1 cytokines classically activate MΦs, in case of the liver resident ones such as Kupffer cells as well, that in turn recruit neutrophils and other leukocytes by expression of various immune mediators [2, 45]. As a consequence, an acute inflammation is initiated which induces overproduction of toxic radicals [2]. Expression of inducible nitric oxide synthase (iNOS) is enhanced via activation of proinflammatory cytokines such as IFN-γ, IL-1β and TNF-α that causes the release of nitrite free radicals, however may perpetuates parenchymal damage by inducing apoptosis [46]. A specific hydatid antigen (antigen 5) has been shown to induce production of nitric oxide and IFN-γ in PBMC cultures derived from hydatid patients [47]. Immunohistochemical detection of iNOS in liver biopsies and its significant levels in the sera as well as in hydatid fluid have been also reported in CE patients [47]. On the other hand, Th2-like cytokines such as IL-4, IL-5 and IL-13 are predominant in immune responses to the chronic phase of infection which induce alternative activation of MΦs and upregulate the ADCC response [9]. Conceptually, tissue-dwelling helminths should employ mechanisms by which they can diminish ADCC-related inflammatory responses to prolong their survival within the host tissue [9]. Study showed that some antigenic compounds of the parasite reduce neutrophil activation *in vitro* [22, 48]. Results achieved by the current study also confirmed that CD68^+^ MΦ and MPO^+^ neutrophils weakly infiltrate the liver parenchyma surrounding the cyst in chronic infection. Nonetheless, largely impaired activities of FOXP3^+^ and CD4^+^ cells along with insignificant expression of CTLA4 mRNA in CE biopsies could suggest that underlying mechanisms, by which the parasite mediates the long-lasting suppression of immune responses in the human liver, are probably T cell-independent. It could imply the immune inhibitory properties of parasite antigens that may directly affect the inflammatory cell activation around the pericyst.

In contrast to T cell costimulatory proteins, molecular assay indicated the remarkable levels of CD80 and CD86 mRNA in the CE biopsies as well as in the other sample groups. These molecules are constitutively expressed on the surface of DCs, as professional antigen presenting cells, but can be also seen on MΦs and B cells upon stimulation [49]. Resting DCs express CD86 at higher level than CD80, but the expression of both molecules is enhanced following activation [49]. They share the same ligands on the surface of T cells and it is conventionally believed that CD80/CD86-CD28 engagement transduces the secondary activation signal. Expression of CTLA4 is augmented on activated T cells and this molecule binds to the same ligands with higher affinity and delivers inhibitory signal [49]. Recent studies, however, have indicated that signal transduction through CD86 may enhance the immune response but CD80 conducts immunosuppressive signals [49, 50]. Therefore, the imbalance between CD86 and CD80 can be an indicator for pathogenesis of inflammatory diseases, as it has been described elsewhere [51]. In CE and CH samples, the expression of CD80 and CD86 at the mRNA level was positively correlated, indicating the continuous activation of antigen presenting function and a chronic inflammation. Perhaps, the absence of CD1a^+^ cells in the pericystic adventitia represented the participation of specific DC phenotypes in hydatid-induced inflammation of the liver. In human, heterogeneous lineages of DCs comprising CD1a^+^ and CD1a^−^ subsets have been identified in different compartment such as peripheral blood [52]. Myeloid DCs express CD1a in response to type 1 cytokines, but evolve into CD1a^−^ subtypes in absence of exogenous mediators *in vitro* [53]. CD1a^+^ DCs produce significant amount of IL-12 and can polarize naïve T cell to Th1 [54]. The involvement of DCs in immunopathology of chronic CE has not been thoroughly studied yet. The only available data show the ability of parasite antigens to induce alternative differentiation of murine and human DCs *in vitro*, which results in overproduction of CD86 and downregulation of CD1a and their enhanced ability to activate Th2 or T_reg_ responses [23, 55–57]. Altogether, results of the present study could also imply the crucial role of DCs in the tissue-specific immune response in human chronic CE of the liver. These results may also highlight the contribution of distinctive DC subtypes in the hydatid-induced chronic inflammation of the liver, which needs further investigation.

Finally, an excessive accumulation of α-SMA^+^ cells and consequent fibrosis in the immediate vicinity of hydatid cyst in all CE samples, showed the presence of continous wound-healing processes. It could be challenged by the inefficient activity of inflammatory cells around the pericyst, which was detected in this study. Additionally, a contribution of B cells to the formation of liver fibrosis cannot be excluded [58, 59]. It is further future issue, whether hydatid antigens can directly mediate liver fibrosis, as it has been implied in other helminth infections such as schistosomiasis [60].

## Conclusion

This is the first comprehensive analysis of the inflammatory infiltrates of the chronic echinococcal infection of human liver. Our study indicated an attenuated cell-mediated immunity and diminished activation of various T cell phenotypes in infected liver. The complete absence of CD1a + DC cells and the presence of a significant amount of B cells could hallmark the essential role of APCs, more likely DCs with CD1a^−^ phenotype [28], in regulation of homeostatic changes and tissue-dependent responses [29] that result in distinctive immunopathology of CE in the human liver. Further studies with larger number of samples are necessary to clarify the function of recruited or resident effector cells involved in the liver immune reactions against chronic CE.

## Abbreviation

AgB: Antigen B
APC: Antigen presenting cell
CE: Chronic echinococcal infection
CH: Chronic hepatitis
DAB: Diaminobenzidine
DC: Dendritic cell
IHC: Immunohistochemistry
MPO: Myeloperoxidase
PCR: Ploimerase chain reaction
SD: Standard deviation
SH: Steatohepatitis
SMA: Smooth muscle actin
